# Overexpression of PD-L1 is an Independent Predictor for Recurrence in HCC Patients Who Receive Sorafenib Treatment After Surgical Resection

**DOI:** 10.3389/fonc.2021.783335

**Published:** 2022-01-18

**Authors:** Yifei Tan, Qing Xu, Zhenru Wu, Wei Zhang, Bo Li, Bohan Zhang, Xi Xu, Bo Zhang, Ke Yan, Jiulin Song, Tao Lv, Jian Yang, Li Jiang, Yujun Shi, Jiayin Yang, Lunan Yan

**Affiliations:** ^1^ Department of Liver Surgery, Liver Transplantation Center, West China Hospital of Sichuan University, Chengdu, China; ^2^ Institute of Clinical Pathology, Key Laboratory of Transplant Engineering and Immunology, NHC, West China Hospital, Sichuan University, Chengdu, China; ^3^ Guangdong Provincial Key Laboratory of Malignant Tumor Epigenetics and Gene Regulation, Sun Yat-Sen Memorial Hospital, Sun Yat-Sen University, Guangzhou, China; ^4^ West China School of Public Health, Sichuan University, Chengdu, China

**Keywords:** hepatocellular carcinoma, liver resection, sorafenib, PD-L1, PD-1

## Abstract

**Objective:**

The predicting values of programmed cell death protein 1 (PD-1) and programmed death-ligand 1(PD-L1) were unclear in Hepatocellular carcinoma (HCC) patients who receive sorafenib treatment after curative hepatic resection.

**Methods:**

We retrospectively enrolled HCC patients who received adjuvant sorafenib treatment after curative resection (N = 154), and patients had resection alone (N = 312). Immunohistochemistry was used to assess expression of PD-1 on tumor infiltration immune cells and PD-L1 on HCC cells. Cox proportional hazard models were used to explore association between clinicopathological factors and risk of tumor recurrence.

**Results:**

No significant difference was detected in RFS (p = 0.542), or OS (p = 0.542) between the resection and sorafenib group and resection alone group. In the 154 patients who received adjuvant sorafenib, expression of PD-1 or PD-L1 was not significantly associated with long-term outcomes. However, in the 122 patients at high risk of postoperative recurrence who had adjuvant sorafenib treatment, characterized by maxim tumor size ≥5 cm, or the presence of macro- or micro-vascular invasion, patients with PD-L1 overexpression (≥3.0) had significantly worse RFS (p = 0.021), and overexpression of PD-L1 (HR: 1.88, 95%CI: 1.18–2.99, p = 0.008) was identified as an independent risk factor associated with unfavorable RFS.

**Conclusion:**

Overexpression of PD-L1 serves as an independent predictor of recurrence in HCC patients at high risk of relapse who received adjuvant sorafenib treatment after curative resection.

## Introduction

Hepatocellular carcinoma (HCC) is the third leading cause of cancer death worldwide, and more than 800 thousand new cases were diagnosed in 2018 alone (1). Curative liver resection remains as the first line therapy for HCC, however, tumor recurrence occurred in approximately 70% patients after curative treatment or transplantation, resulted in 5-year survival rates ranging from 40.0 to 71.9% (2, 3) depending on tumor stage.

As the first approved systemic drug for advanced HCC, sorafenib prolonged the median time to radiologic tumor progression and survival by about three months in patients with advanced HCC (4, 5). Anti-tumor activity of sorafenib mainly involves inhibition of tumor cell proliferation and angiogenesis by blocking the RAF/MEK/ERK pathway (6, 7), and sorafenib was expected to reduce postoperative HCC recurrence that resulted from growth of unrecognized residual tumor cells or circulating tumor cells. Based on an orthotopic mouse model, sorafenib was demonstrated to suppress HCC recurrence after resection by blocking activated oncogenic kinase pathways (8). Whereafter, several studies (9–12) were conducted to explore the effect of sorafenib as an adjuvant therapy after surgery in HCC patients, but ended up with inconclusive results. However, most studies were based on quite small sample size and were retrospectively conducted that selection bias was inevitable though case-matching was performed in some studies (12, 13). On the other hand, the only phase III trial of sorafenib as adjuvant therapy, the STORM trial, revealed no difference in recurrence free survival (RFS) after curative resection or ablation between sorafenib and placebo arm in early HCC (14).

It should be noted that HCC patients with macrovascular invasion, in which sorafenib was reported to improve RFS (11, 12), were not included in the STORM trail. In addition, histological features and expression of biomarkers were not available in patients who underwent ablation. It is possible that effect of adjuvant sorafenib varies with different histological tumor characters or expression of certain biomarkers which involve in signaling pathways related to tumor progression. In fact, several biomarkers have been revealed as potential determinant for primary resistance to sorafenib in HCC based on mouse model or cell line, such as epidermal growth factor receptor (EGFR) (15).

The mechanism of immune escape promoting tumor progression has been well understood in recent years, and PD-1 (programmed cell death protein 1) and PD-L1 (programmed death-ligand 1), the important immune checkpoints which facilitate tumor evasion, is of particular interest. Overexpression of PD-L1 was associated with increased HCC aggressiveness (16) and risk of tumor relapse in patients who receive curative resection (17, 18). In addition, single-cell profiling has recently uncovered improved evasion ability of recurrent HCC cells in comparison to primary HCC, in which PD-L1 suppressed antigen presentation efficacy of dendritic cells (19). Interestingly, recurrent malignant cells exhibited higher expression of PD-L1 compared with primary HCC (19), and similar upregulation occurred in HCC cells (20) after sorafenib treatment.

Moreover, overexpression of PD-L1 promotes aggressiveness of sorafenib-resistant HCC cells by facilitating EMT *via* the PI3K/Akt pathway (21). On the other hand, treatment targeting PD-1/PD-L1 showed antitumor activity in sorafenib-treated orthotopic HCC model (20) and sorafenib-resistant cell lines (22). While the biochemical rationale for the effect of PD-1/PD-L1 in sorafenib-treated HCC has been at least partially described, the role of this important immune checkpoint is unclear in patients who receive adjuvant sorafenib. In this study, we compared long-term outcomes of patients who received adjuvant sorafenib after hepatic resection with matched patients who underwent curative hepatectomy only. Besides, the effect of PD-1/PD-L1 expression in adjuvant sorafenib-treated HCC patients was learned in general and in specific patients at relative high risks of tumor recurrence.

## Methods

### Patients and Design

We retrospectively included HCC patients who underwent curative liver resection in the West China Hospital of Sichuan University between January 2014 and June 2019, while all patients were prospectively followed. The inclusion criteria were as follows: 1) adult patients with a pathologically confirmed the first diagnosis of HCC; 2) did not received any neoadjuvant anti-tumor therapy. Demographic and clinicopathological information were derived from the electric database of the hospital. The study was conducted in accordance with principles of the Declaration of Helsinki, and the protocol was approved by the Ethics Committee of the West China Hospital of Sichuan University West China Hospital (2019–482).

Generally, sorafenib was recommended if a patient was defined as having a high risk of postoperative recurrence based on pathological findings: a maximum tumor size of ≥5 cm, or three or more tumors, or presence of microvascular invasion (MVI) or macrovascular invasion. Patients were also eligible if with a high preoperative AFP level (>1,210 ng/ml) or serum AFP did not decrease to a satisfying level at the first follow-up test. In our center, all patients received comprehensive assessment before beginning sorafenib treatment, namely, general condition, tumor features, and blood test. Patients considering sorafenib therapy accepted CT or MRI scan regardless of ultrasound results which was routinely conducted at one month after surgical resection, and a signed, informed consent was required. For both groups (with or without sorafenib therapy), the exclusion criteria were: 1) R1 resection at histology; 2) confirmed recurrence by radiological scan (CT/MRI) at one month after surgery or before sorafenib therapy; 3) systemic treatment other than sorafenib until recurrence occurred; and 4) follow-up or tumor specimen unavailable. For patients who took adjuvant sorafenib, those experienced discontinuity of sorafenib therapy within three months due to severe adverse effect were also excluded.

Sorafenib therapy generally began with 400 mg, twice a day, and therapy interruption or dose reduction were determined when adverse effect occurred. An initial dose of 200 mg, twice a day due to unsatisfying general condition or a dose reduction to 400 mg every other day was acceptable, but those with further dose reduction were excluded from the study.

### Follow-Up

A routine blood test for liver function, serum a-fetoprotein (AFP), and an abdominal ultrasound were performed one month after surgery. Patients were followed in the outpatient clinic for blood count, hepatic function, AFP and protein induced by vitamin K antagonist-II (PIVKA-II), and ultrasound test every three months in the first postoperative year and every six months thereafter. An enhanced CT or MRI was applied in any patient with positive finding of AFP/PIVKA-II level or abdominal ultrasound. RFS was calculated by the interval between the date of liver resection and recurrence or death by any cause, or the date of last available follow-up.

### Expression of PD-1 and PD-L1

Histopathology briefly, the paraffin tissue sections were dewaxed and hydrated, followed by antigen retrieval. Subsequently, the tissue slides were incubated with primary antibodies using rabbit anti-human PD-1 polyclonal antibody (5 μg/ml, cat # PA5-20351; Invitrogen; Thermo Fisher Scientific, Inc., Waltham, MA, USA.) or mouse anti-human PD-L1 monoclonal antibody (5 μg/ml cat # 14-5983-82; Invitrogen) at 4°C overnight, followed by incubation with secondary antibodies (cat # K5007; Dako). Staining was performed with DAB and counterstained with hematoxylin. Two senior pathologists who were blinded to the clinical data independently selected five non-overlapping and discontinuous regions to calculate the mean for statistical analysis. The numbers of PD-1 and PD-L1 cells were quantified at ×400 (0.0484 mm^2^). Variations in the results beyond a range of 5% were reassessed, and a consensus decision was made. Results of PD-1 and PD-L1 expression were presented as the proportion of PD-L1 + tumor cells (PD-L1 + tumor cells/total tumor cells), PD-1 + tumor infiltrating immune cells (PD-1 + immune cells/total immune cells), and the cut-off values for PD-1 and PD-L1 overexpression were determined by x-tile software based on recurrent events.

### Public Data Source Acquisition

The RNA-seq transcriptome data and corresponding clinical information of HCC samples were downloaded from The National Cancer Institute Genomic Data Commons (NCI-GDC) (https://gdc.cancer.gov/). A total of 110 HCC cases were included for subsequent analysis. The TCGA HCC cohort was stratified into a PD-L1^high^ group and PD-L1^low^ group based on the median value of the expression of PD-L1 (0.30001).

### Statistical Analysis

A 1:1 propensity score match (PSM), with a caliper of 0.05, was performed to overcome potential bias of covariates between patients with and without sorafenib therapy. Factors used for PSM included age, gender, maxim tumor size, number of tumors (solitary/multiple), and Child–Pugh grade. Continuous data was presented with mean and standard, and was compared using Student’s t test while the Chi-square or Fisher’s exact test was applied for categorical data. Kaplan–Meier curves were depicted to plot cumulative RFS and overall survival (OS), and a log-rank test was performed to determine significance. In regards to PD-1 and PD-L1, x-tile software was used to identify optimal cut-off value based on recurrence events with corresponding time. The association between clinicopathological factors and risk of tumor recurrence were analyzed using Cox proportional hazard models, and hazards ratios (HRs) with corresponding 95% confidence intervals (CI) were estimated. Potential and significant factors determined in the univariate analysis were further included in the multivariate-adjusted models. A two-tailed p-value of lower than 0.05 was determined significant difference for all analysis. Statistics were performed with IBM SPSS Statistics version 26.0.0.0 (IBM Corp., Armonk, NY, USA).

## Results

During the study period, 539 patients were initially included, of which 356 patients received curative resection only while the other 183 received resection and adjuvant sorafenib treatment. In the adjuvant sorafenib group, 29 patients were excluded due to R1 resection, recurrence one month after surgery or prior to sorafenib intake, other systemic therapy or follow-up or tumor specimen unavailable, leaving 154 patients for final analysis. A total of 312 patients were eligible to the non-sorafenib group (**Figure S1**). As shown in **Table 1**, the two groups differed significantly in terms of age, maxim tumor size, number of tumors (solitary/multiple), macrovascular invasion, BCLC stage, TNM stage, ALT, ALB and Child–Pugh grade. After PSM was applied, 131 patients were left in each group, and no significant difference was found in baseline characters between the two matched groups.

**Table 1:** Characters of patients concerning postoperative sorafenib therapy before and after PS match.

	Before match			After match		
	Sorafenib (−)	Sorafenib (+)	p	Sorafenib (−)	Sorafenib (+)	p
	N = 312	N = 154		N = 131	N = 131	
age	50.7 + 11.6	53 + 11.9	0.048	52.7 + 10.9	52.4 + 12	0.825
age ≥55 (%)	121 (39)	63 (40.9)	0.762	55 (42)	51 (38.9)	0.706
Male (%)	269 (86.2)	142 (92.2)	0.067	113 (86.3)	120 (91.6)	0.237
Max tumor size	7.1 + 3.9	6.3 + 3.6	0.032	6.1 + 3.2	6.4 + 3.6	0.516
size ≥5 cm (%)	219 (70.4)	94 (61)	0.046	77 (58.8)	82 (62.6)	0.613
Multiple tumors (%)	56 (17.9)	40 (26)	0.051	33 (25.2)	37 (28.2)	0.675
Vascular invasion (VI)						
Macro-VI (%)	32 (10.3)	29 (18.8)	0.008	13 (9.9)	20 (15.3)	0.267
Micro-VI (%)	114 (36.5)	70 (45.5)	0.098	50 (38.2)	57 (43.5)	0.451
BCLC						
0/A	243 (77.9)	87 (56.5)	<0.01	84 (64.1)	87 (66.4)	0.404
B	37 (11.9)	38 (24.7)		27 (20.6)	31 (23.7)	
C	32 (10.3)	29 (18.8)		20 (15.3)	13 (9.9)	
TNM Stage (I/II/III)	163/89/60	54/45/55	<0.01	60/35/36	49/40/42	0.386
TB	16.6 +23.8	17.6+ 10.5	0.612	16.4 + 14.7	16.2 + 7.9	0.889
ALT	48.6 + 38.6	60.9 + 89.4	0.038	49.6 + 40	53.9 + 63.2	0.520
AST	51.5 + 40.1	70.6 + 132.8	0.083	48.6 + 32.2	66.6 + 133.7	0.135
ALB	41.5 + 4.7	42.8 + 6.3	0.025	41.4 + 4.4	42.3 + 6.3	0.169
Cirrhosis	182 (58.7)	94 (61)	0.617	72 (55)	76 (58)	0.707
Poor Differentiation (%)	146 (46.8)	61 (39.6)	0.141	63 (48.1)	54 (41.2)	0.320
HBV (%)	283 (90.7)	140 (90.9)	0.597	115 (87.8)	118 (90.1)	0.694
Child–Pugh grade A (%)	305 (97.8)	144 (93.5)	0.033	128 (97.7)	127 (96.9)	1.000
AFP ≥200 (%)	147 (47.3)	65 (42.2)	0.548	56 (42.8)	57 (43.5)	0.801

BCLC, Barcelona Clinic Liver Cancer; TNM, tumor-node-metastasis; TB, total bilirubin; ALT, alanine transaminase; AST, aspartate transaminase; ALB, albumin; HBV, hepatitis B virus; AFP, alpha fetoprotein.

### Matched Patients With or Without Adjuvant Sorafenib Had Similar Outcomes

**Figure 1:**
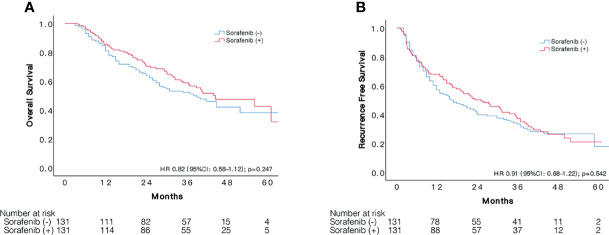
Comparison of survival between patients with sorafenib treatment after resection and those had resection alone. Kaplan–Meier curves of OS **(A)** and RFS **(B)** of patients with and without adjuvant sorafenib treatment after liver resection.

The median follow-up time was 35.9 (+16.2) months in the 131 pairs of patients. Overall median RFS period was 25.3 (95%CI: 16.8–33.9) months for sorafenib group and 17.0 (95%CI 10.8–23.2) months for the other. Patients with and without adjuvant sorafenib therapy had similar cumulative recurrence rate (HR:0.92, 95%CI 0.68–1.22, p = 0.542), resulted in 1, 3, and 5-year RFS of 67.9, 38.4, 21.1% and 60.2, 34.2, 17.8%, respectively (**Figure 1**). Likewise, sorafenib intake was not associated with better OS (HR:0.82, 95%CI 0.58–1.15, p = 0.542), resulted in 1, 3, and 5-year OS of 85.5, 58.9, and 42.6% in sorafenib group compared to 84.7, 52.2, and 38.2% in the non-sorafenib group.

### Insignificant Effect of PD-1/PD-L1 Status in Unselected Patients With Sorafenib Treatment

**Figure 2:**
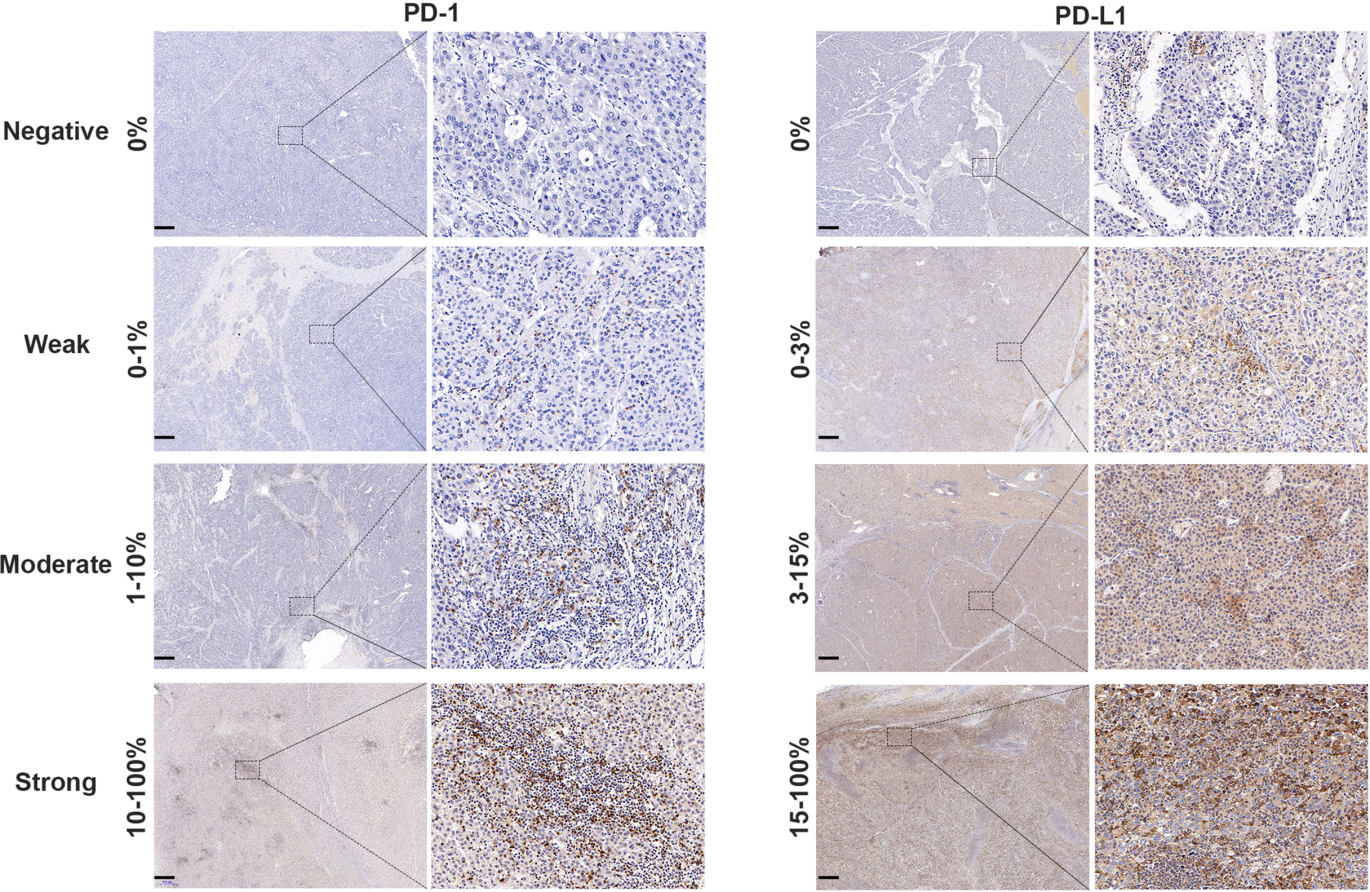
PD-1 and PD-L1 expression in HCC tissue samples. Representative micrographs of PD-1 and PD-L1 expression within tumor (scalebar, 500 μm). The PD-1 status of tumor tissues was judged as negative (0%), weak (0–1%), moderate (1–10%) or strong (10–100%) according to the positive proportion of PD-1 in immune cells. The PD-L1 status of tumor tissues was classified as negative (0%), weak (0–3%), medium (3–15%) or strong (15–100%) based on the positive proportion of PD-L1 in tumor cells.

**Figure 3:**
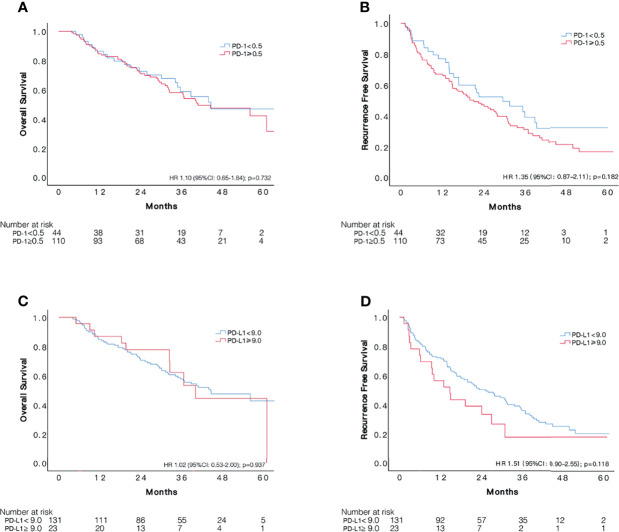
Comparisons of survival among patients with different status of PD-1 and PD-L1 expression in 154 unselected HCC patients who received adjuvant sorafenib. Kaplan–Meier curves of OS and RFS according to expression of PD-1 **(A, B)** and PD-L1 **(C, D)**.

The cut-off value of PD-1 was 0.5 for the 154 patients with sorafenib therapy according to x-tile software, of which 110 patients were defined as overexpressing PD-1. Examples of PD-L1 expression in HCC tissues are shown in **Figure 2**. However, no significant difference was detected in RFS (HR 1.35, 95%CI: 0.87–2.11, p = 0.182) between the two groups. The median RFS in the PD-1 lower expressed group was 29.6 (95%CI:15.1–44.1) months compared to 21.2 (95CI: 14.3–28.1) months in the PD-1 overexpressed group. Comparable OS (HR 1.10, 95%CI: 0.65–1.84, p = 0.729) was observed in the two groups. In terms of PD-L1, patients with PD-L1 higher than 9.0 was defined as PD-L1 overexpressed. PD-L1 overexpressed and lower expressed patients had insignificantly different recurrence rate (HR 1.51, 95%CI: 0.90–2.55, p = 0.118), with a median RFS of 14.8 (95%CI: 7.1–22.4) months and 25.3 (95%CI: 17.1–33.5) months, respectively. On the other hand, OS in patients with overexpression of PD-L1 did not differ from that in patients with lower expression of PD-L1 (HR 1.02, 95%CI: 0.53–2.00, p = 0.937). Cumulative RFS and OS curves according to PD-1 and PD-L1 status are depicted in **Figure 3**.

### PD-L1 Overexpression Was Associated With Unfavorable RFS in 122 HCC Patients at High Risks of Recurrence

One hundred and twenty-two patients met our definition of a high risk of HCC recurrence, of which 86 (70.5%) subjects suffered recurrence during follow-ups. According to x-tile software, optimal cut-off value for PD-1 was 1.0, assigning 86 and 36 patients, respectively, to PD-1 overexpressed and control group. PD-1 overexpressed patients had relatively low RFS compared to the control group, however, the difference was not significant (HR1.31, 95%CI: 0.81–2.14, p = 0.267). The median RFS time in patients with lower expression of PD-1 was 29.6 (95%CI: 14.9–44.3) months and 20.0 (95%CI: 11.4–28.6) months in patients with overexpression, respectively. Meanwhile, the comparison of OS between the two groups indicated no difference, resulted in a HR of 0.94 (95%CI: 0.54–1.64, p = 0.819).

**Figure 4:**
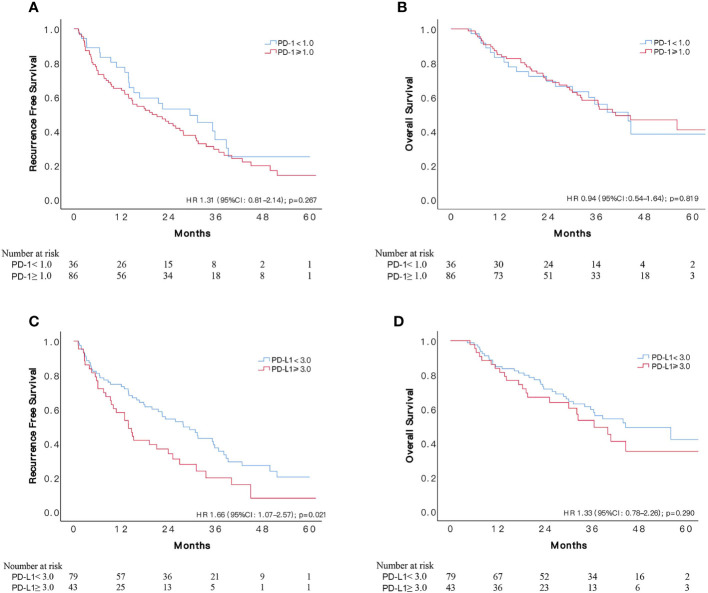
Comparisons of survival among patients with different status of PD-1 and PD-L1 expression in 122 patients at high risk of recurrence who received adjuvant sorafenib. Kaplan–Meier curves of OS and RFS according to expression of PD-1 **(A, B)** and PD-L1 **(C, D)**.

A PD-L1 higher than 3.0 was determined as overexpression of PD-L1 based on recurrent events and corresponding time, assigning 43 and 79 patients, respectively, to PD-L1 overexpressed and lower expressed group. During a median follow-up period of 22.6 months, 34 (79.1%) and 52 (65.8%) recurrences occurred in PD-L1 overexpressed and lower expressed group, respectively. Patients with overexpressed PD-L1 status were observed with significantly worse cumulative recurrence rate (HR 1.66, 95%CI: 1.07–2.57, p = 0.021), with an overall median RFS time of 14.0 (95%CI: 9.6–18.4) months compared to 28.0 (95%CI: 18.8–37.2) months in patients with lower expression of PD-L1. About three quarters of recurrences were intrahepatic in both groups, and transcatheter arterial chemoembolization (TACE) was the most commonly used treatment for recurrence. No significant difference was found concerning recurrence site, number and salvage treatment between the two groups (**Table S1**). The two groups had similar OS during follow-ups, resulted in a HR of 1.33 (95%CI: 0.78–2.26, p = 0.290). Cumulative RFS and OS according to PD-1 and PD-L1 status are depicted in **Figure 4**. We also applied 3.0 as cut-off value for PD-L1 in the 154 unselected HCC patients, and the difference in RFS remained insignificant (p = 0.254) between the two groups (**Figure S2**), with a median RFS of 27.6 (95%CI: 19.7–35.5) months in patients with lower expression of PD-L1 (N = 100) and 15.0 (95%CI: 7.5–22.5) months in the PD-L1 overexpressed group (N = 54).

Among the 122 patients with high risk of recurrence, patients with overexpression and lower expression of PD-L1 had comparable baseline characters, namely, age, gender, tumor factors, liver function, and presence of cirrhosis (**Table 2**). However, poor differentiation was more frequently identified in patients with lower expression of PD-L1 (p = 0.037).

**Table 2:** Comparison of baseline characters of 122 patients with high risk of recurrence concerning PD-L1 level.

	PD-L1 (−)	PD-L1 (+)	p
	N = 79	N = 43	
Age	52.6 + 12.2	52.2 + 12.2	0.857
Age ≥55 (%)	31 (39.2)	17 (39.5)	1.000
Male (%)	74 (93.7)	38 (88.4)	0.321
Max tumor size	7.1 + 2.9	7.5 + 4.2	0.500
Size ≥5 cm (%)	63 (79.7)	31 (72.1)	0.372
Multiple tumors (%)	20 (25.3)	17 (39.5)	0.148
Vascular invasion (VI)			
Macro-VI (%)	16 (20.3)	13 (30.2)	0.267
Micro-VI (%)	49 (62)	21 (48.8)	0.183
BCLC			
0/A	40 (50.6)	16 (37.2)	0.306
B	23 (29.1)	14 (32.6)	
C	16 (20.3)	13 (30.2)	
TNM Stage (I/II/III)	18/28/33	11/13/19	0.838
TB	17.5 + 9.9	17.2 + 10.7	0.859
ALT	64.6 + 79.7	53.5 + 63.8	0.433
AST	75.4 + 94	79.6 + 210.2	0.879
ALB	42.4 + 7.4	42.3 + 5.3	0.964
Cirrhosis	49 (62)	28 (65.1)	0.845
Poor Differentiation (%)	44 (55.7)	15 (34.9)	0.037
HBV (%)	75 (94.9)	36 (83.7)	0.333
Child–Pugh grade A (%)	6 (7.6)	3 (7)	1.000
AFP ≥200 (%)	36 (45.6)	19 (44.2)	1.000

PD-L1, programmed death-ligand 1; BCLC, Barcelona Clinic Liver Cancer; TNM, tumor-node-metastasis; TB, total bilirubin; ALT, alanine transaminase; AST, aspartate transaminase; ALB, albumin; HBV, hepatitis B virus; AFP, alpha fetoprotein.

Increased risk of tumor recurrence was associated with maxim tumor size of ≥5 cm, presence of macro-vascular invasion, serum AFP ≥200 ng/ml and overexpression of PD-L1. Presence of macro-vascular invasion had a higher HR (2.41, 95%CI: 1.52–3.84) than other risk factors in the univariate analysis, followed by maxim tumor size of ≥5 cm (HR 1.90, (95%CI: 1.12–3.24). Subjects with MVI tended to suffer worse RFS that those without MVI, however, the difference was not significant (HR 1.50, 95%CI: 0.97–2.34), similarly with multiple tumors (HR 1.44, 95%CI: 0.92–2.25) and overexpression of PD-1 (HR 1.31, 95%CI: 0.81–2.14). Neither poor differentiation nor liver cirrhosis was found to be associated with increased risk of recurrence, with HRs of 1.15 (95%CI: 0.75–1.76) and 1.28 (95%CI: 0.82–2.00), respectively.

In the multivariate hazards model, maxim tumor size of ≥5 cm, presence of macro-vascular invasion and PD-L1 ≥3.0 remained significantly associated with increased risk of recurrence, while serum AFP ≥200 ng/ml (HR 1.42, 95%CI: 0.91–2.20) was not an independent risk factor. Though MVI was not a significant factor in univariate analysis, it was associated with an increased risk of RFS in the multivariate model, with a HR of 1.61(95%CI: 1.02–2.55). Both maxim tumor size of ≥5 cm (HR 2.13, 95%CI: 1.23–3.69) and macro-vascular invasion (HR 2.08, 95%CI: 1.29–3.37) were related with an approximately two-fold risk of recurrence, while a 1.88-fold risk (95%CI: 1.18–2.99) was observed in patients with overexpression of PD-L1 (**Table 3**). Based on the independent risk factors, a RFS prognostic nomogram was established according to the results of multivariate analysis (**Figure 3**).

**Table 3:** Univariate and multivariate analysis of risk factors of tumor recurrence in 122 patients.

	Univariate		Multivariate	
	HR (95%CI)	P	HR (95%CI)	p
Age ≥55	0.88 (0.57–1.36)	0.576		
male	0.57 (0.28–1.14)	0.111		
Size ≥ 5cm	1.9 (1.12 –3.24)	0.018	2.13 (1.23–3.69)	0.007
Multiple tumors	1.44 (0.92–2.25)	0.114		
Vascular invasion				
Macro-VI	2.41 (1.52–3.84)	<0.001	2.08 (1.29–3.37)	0.003
Micro-VI	1.5 (0.97–2.34)	0.071	1.61 (1.02–2.55)	0.042
Poor differentiation	1.15 (0.75–1.76)	0.516		
Child B	1.1 (0.48–2.50)	0.823		
AFP ≥200	1.61 (1.04–2.49)	0.032	1.42 (0.91–2.20)	0.122
PD-L1 ≥3.0	1.66 (1.07–2.57)	0.023	1.88 (1.18–2.99)	0.008
PD-1 >1.0	1.31 (0.81–2.14)	0.269		
Liver cirrhosis	1.28 (0.82–2.00)	0.281		

HRs, hazards ratios; CI, confidence intervals; AFP, alpha fetoprotein; PD-L1, programmed death-ligand 1; PD-1, programmed cell death protein 1.

### Bioinformatic Analysis

To further explore the potential molecular mechanism between PD-L1 and sorafenib, we analyzed the expression correlation between PD-L1 and sorafenib related target molecules by Heatmap. We found that Flt-3, PDGFR β, PD-1 were positively correlated with the expression of PD-L1 (**Figure 4A**). In addition, we used KEGG gene set (C2) to analyze TCGA HCC data set by GSEA. Cog. V6.2. Symbol). According to the results, the high expression of PD-L1 was negatively associated with immune related signals, antigen processing and presentation, T cell receptor signaling pathway, natural killer cell mediated cytotoxicity, Toll like receptor signaling pathway and cytokine–cytokine receptor interaction. We also indicated that the high expression of PD-L1 was negatively correlated with JAK-STAT signal, which was consistent with the report that sorafenib inhibited proliferation and invasion of HCC through JAK-STAT pathway (23) (**Figure S4B**).

## Discussion

We here present the first evidence that expression of PD-L1 is an independent predictor of postoperative recurrence after hepatic resection in patients at high risk of HCC relapse who received adjuvant sorafenib treatment. On the other hand, we found that adjuvant sorafenib treatment after curative resection was not correlated with improved RFS or OS.

The need for effective adjuvant therapy for HCC remains highly unmet as the STORM trial failed in early-stage HCC (14). However, HCC patients in China were at relative younger age and advanced stage when diagnosed (24), as most cases were caused by hepatitis B infection which is generally acquired at birth or early life. Our experience (24) suggest liver resection as the first choice for patients with intermediate and advanced HCC based on strict selection of liver function and residual liver volume. Therefore, large tumor size, multinodular or the presence of macrovascular invasion should not be contraindication for hepatic resection, and adjuvant sorafenib therapy is an option. As a fact, protective effect of adjuvant sorafenib were observed in advanced HCC cases (11, 12), supported by a recent meta-analysis (25). However, we found that a combination of resection and adjuvant sorafenib did not confer favorable RFS or OS over resection alone, which was consistent with the STORM trail (14) and another report (10). The anti-tumor activity of sorafenib mainly involves inhibition of angiogenesis and malignant cellular proliferation, however, proliferative function signals is upregulated in primary HCC but not in relapsed HCC (19). This is a potential explanation for the success of sorafenib in primary advanced HCC (4) and the absence of benefit in preventing postoperative recurrence (14).

While it is difficult to draw a firm conclusion on the benefit of adjuvant sorafenib, potential predictors for outcomes following adjuvant sorafenib had been explored. Evidence showed that overexpression of TNF-α (26) and two systemic inflammation parameters, the neutrophil-to-lymphocyte ratio (NLR) and gamma-glutamyl transferase (GGT) (27), were effective prognostic factors in sorafenib-treated patients. In addition, the biomarker companion study of the STORM trial, namely, the BIOSTROM study (28), demonstrated that phosphorylated extracellular signal-regulated kinase (pERK) was independently associated with unfavorable RFS. As a critical factor involved in angiogenesis and apoptosis, pERK enhanced proliferation while leading to poor differentiation by activating MAPK pathway, a key inhibiting target of sorafenib. In the current study, we identified overexpression of PD-L1 (≥3.0), larger (≥5 cm) tumor size (13), presence of macro-vascular invasion (9, 26, 27) and MVI (28) as predictors of RFS in HCC patients who received adjuvant sorafenib after resection, echoing previous studies. While tumor size and vascular invasion were routinely studied in resected HCC tissue, additional evaluation of PD-L1 expression is believed to be of prognostic value in patients considering adjuvant sorafenib therapy. If upregulated PD-L1 is identified, HCC relapse may not be effectively inhibited by sorafenib alone, and supplemental treatment is needed, such as anti-PD-L1 or TACE.

In a previous study where adjuvant sorafenib was not applied, high expression of PD-L1 was found to be independently associated with increased risk of postoperative tumor relapse (17). The ligation of PD-L1 to PD-1 promotes apoptosis of activated tumor-reactive T cells which result in immunosuppression and tumor progression (29, 30). Additionally, recurrent HCC showed a higher proportion of PD-L1+ malignant cells than primary HCC, and competitive binding of PD-L1 to CD80 inhibited antigen presentation which further led to compromised anti-tumor immunity (19).

In the unselected HCC patients who received sorafenib treatment after resection, overexpression of neither PD-1 nor PD-L1 was related to unfavorable RFS, though tended to. However, in the subgroup analysis of 122 patients with high risk of recurrence, PD-L1 overexpressed group showed worse RFS than that of PD-L1 lower expressed group, while PD-1 status was not significantly related to postoperative recurrence rate. Overexpression of PD-L1 has been proven to promote immune evasion and tumor growth in HCC by suppressing tumor-infiltrating lymphocytes, and the mechanism mainly involves reduction of cytotoxic CD8^+^ T cells and expression of cytokines (20, 30). Aberrant upregulation of PD-L1 was observed in sorafenib-resistant HCC cells and overexpression of PD-L1 played an essential role in developing and maintaining resistance to sorafenib which contributed to persistent aggressiveness of HCC cells (31). PD-L1 facilitated EMT and aggressive properties of sorafenib-resistant cells by activating PI3K/Akt pathway which was modulated by expression of Sterol regulatory element-binding protein 1 (SREBP-1) (21). Reversal of EMT was observed after knockdown of PD-L1 and sensitivity of HCC cells to sorafenib was significantly improved (31, 32). More importantly, unlike TNF-α (26), the aforementioned predictor for RFS which was not influenced by sorafenib treatment, PD-L1 expression was significantly upregulated upon exposure to sorafenib and in sorafenib-resistant HCC (20, 31, 33), and also in relapsed HCC (19). In sorafenib-resistant HCC, nuclear factor E2-related factor 2 (NRF-2) promoted expression of PD-L1 by suppressing microRNA-1 (miR-1) which was a tumor-inhibitory microRNA (31). In terms of untreated cells, upregulation of PD-L1 in HCC cells was driven by enhanced expression of TGF-β1 (32) upon exposure to sorafenib, In the present study, 43 (35.2%) out of 122 HCC cases were defined as PD-L1 overexpressed according to x-tile, higher than 25% based on the 75th percentile in the previous study (17) where sorafenib was not applied. The higher proportion of patients with overexpression of PD-L1 might be a reflection of sorafenib-induced upregulation of PD-L1 in HCC. Taking the possible upregulation of PD-L1 and its potential to mediate sorafenib-resistance, it may help improve management strategy by monitoring expression of PD-L1 after adjuvant sorafenib.

Maximum tumor size of ≥5 cm (34, 35), multiple tumors (34), macrovascular/microvascular invasion (34, 36) are robust prognostic factors for HCC relapse, and their absence was applied to define patients with low risk of recurrence in our study. These risk factors are related to presence of residual microscopic lesions and aggressive nature of HCC that makes patients vulnerable to recurrence (37). Compared to patients with high risk, the insignificant correlation between PD-L1 and RFS in patients with low risk of HCC recurrence might be a reflection that PD-L1 or PD-L1-induced EMT requires the abovementioned risk factors to promote regrowth of HCC.

The anti-angiogenesis effect of sorafenib led to hypoxia tumor microenvironment which increased intra-tumoral expression of PD-L1 (20). Anti-PD-1/PD-L1 therapy (22, 31) have been reported to prevent or re-sensitize acquired resistance of HCC cells to sorafenib, though some request additional concomitant agents, such as CXCR4 inhibitors (20). The association between postoperative recurrence and expression of PD-L1, but not PD-1, supports the effect of PD-L1 in regeneration of HCC cells as an inhibitor of antigen presentation *via* PD-L1–CD80 axis (19). In fact, the PD-L1–CD80 interaction even repress PD-1–PD-L1 binding (38), especially when substantial CD80 is available (39), and T cells activation would hardly be inhibited by PD-1/PD-L1 pathway. Besides, CD80 expression on dendritic cells was downregulated by anti-PD-L1, but not by anti-PD-1 treatment (38), suggesting potential benefit of anti-PD-L1 treatment.

While patients with overexpression of PD-L1 suffered poor RFS, insignificant difference was observed in OS between the two groups. The absent of improvement in OS probably attributed to the development of treatment for HCC recurrence, as 41 (47.7%) recurrent cases received potential curative transplantation, resection or ablation in our study, while additional options were available.

The major limitation of the current study involves its retrospective nature, and the recruiting criteria for sorafenib treatment was largely dependent on recommendations from physician and patients’ willingness, thus potential selection bias was inevitable though case-matching was performed. Besides, post-sorafenib HCC tissues were unavailable in most cases, and upregulation of PD-L1 expression (20, 33) by sorafenib treatment was not assessed. All patients were enrolled in mainland China where HBV infection was the predominate cause of HCC, therefore, out findings may not fit in other countries with different etiologic backgrounds of HCC.

### Conclusion

The present study (A) identified overexpression of PD-L1 as an independent predictor for postoperative recurrence in patients at high risk of HCC relapse who received adjuvant sorafenib after curative resection, (B) detected no significant difference in outcomes between patients with or without adjuvant sorafenib treatment. PD-L1 is anticipated to be a potential therapeutic target in patients with overexpression of PD-L1 or sorafenib-resistance, and addition of anti-PD-L1 to sorafenib treatment in the adjuvant setting warrant further validation.

## Data Availability Statement

The raw data supporting the conclusions of this article will be made available by the authors, without undue reservation.

## Ethics Statement

The studies involving human participants were reviewed and approved by the Ethics Committee of West China Hospital. The patients/participants provided their written informed consent to participate in this study.

## Author Contributions

Conception and design: YT, QX, YS, and JiayY. Administrative support: LY, LJ, TL, and JiayY. Collection and assembly of data: YT, QX, ZW, BL, BohZ, XX, and BoZ. (V) Data analysis and interpretation: YT, QX, WZ, and KY; (VI) Manuscript writing: YT and QX. All authors contributed to the article and approved the submitted version.

## Funding

This work was supported by grants from the National Natural Science Foundation of China (81770653) and the 1.3.5 Project for Disciplines of Excellence, West China Hospital, Sichuan University (ZY2017308 and 2020HXFH010)

## Conflict of Interest

The authors declare that the research was conducted in the absence of any commercial or financial relationships that could be construed as a potential conflict of interest.

The reviewer CS declared a shared affiliation, with one of the authors WZ to the handling editor at the time of the review.

## Publisher’s Note

All claims expressed in this article are solely those of the authors and do not necessarily represent those of their affiliated organizations, or those of the publisher, the editors and the reviewers. Any product that may be evaluated in this article, or claim that may be made by its manufacturer, is not guaranteed or endorsed by the publisher.

## Supplementary Material

Supplementary Figure 1 Patients selection flow chart.

Supplementary Figure 2 Comparisons of RFS between patients with PD-L1 (≥3.0) and those with PD-L1 (<3.0) in 154 unselected HCC patients who received adjuvant sorafenib.

Supplementary Figure 3 Recurrence-free survival nomogram of 122 HCC patients who had adjuvant sorafenib. MAVI, macrovascular invasion; MIVI, microvascular invasion; PD-L1, programmed death-ligand 1; RFS, recurrence-free survival.

Supplementary Figure 4 Analysis of correlation between PD-L1 and sorafenib-related target molecules. **(A)** Heatmap showing relation between PD-L1 and other molecules; **(B)** GSEA analysis using KEGG gene set (C2).
