# Supplementary figures and images for: Overexpression of PD-L1 is an Independent Predictor for Recurrence in HCC Patients Who Receive Sorafenib Treatment After Surgical Resection
Source: Front Oncol. 2022 Jan 18;11:783335. doi: 10.3389/fonc.2021.783335 (PMC8804345; doi:10.3389/fonc.2021.783335)

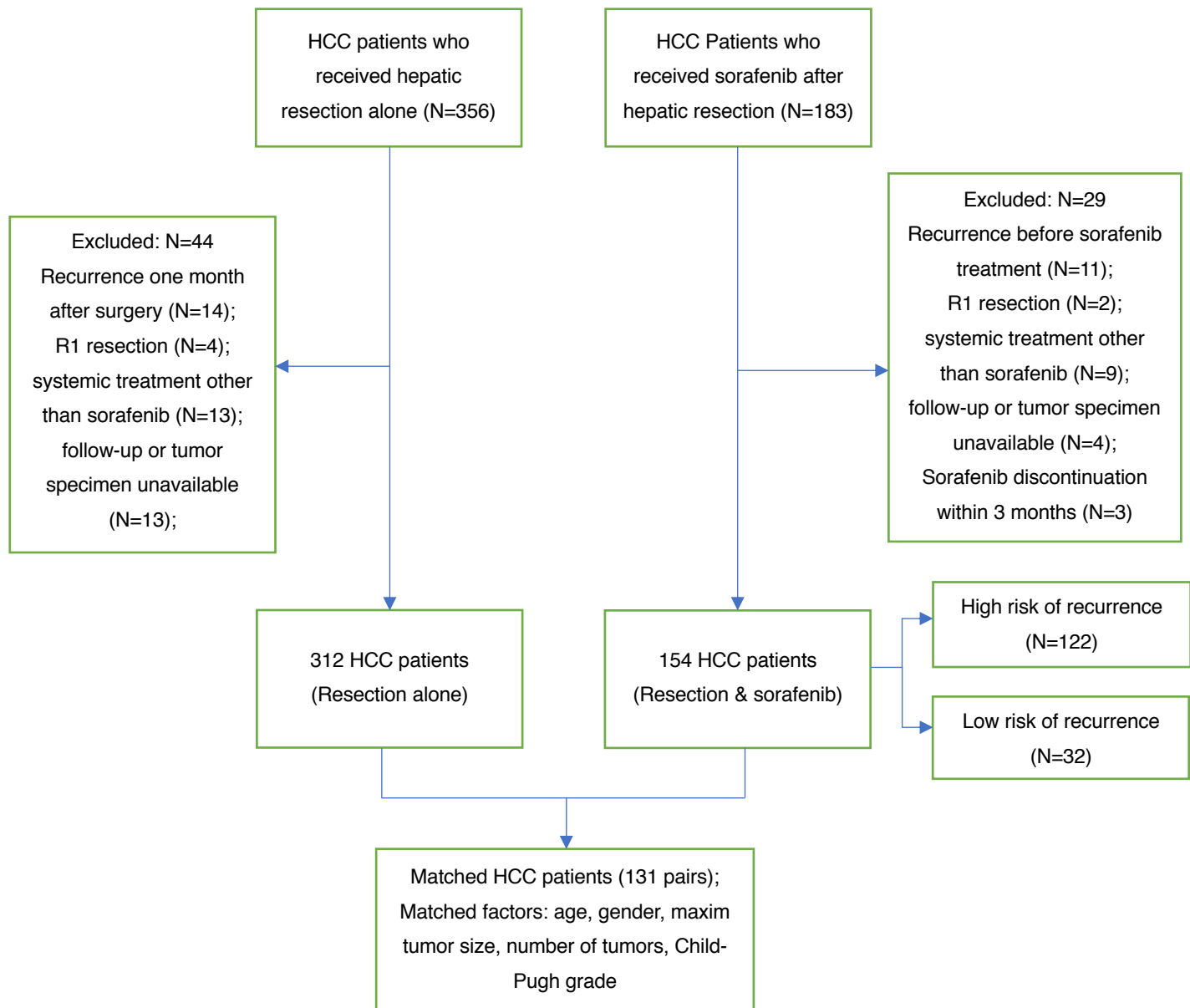

Supplement: Supplementary Figure 1 — Patients selection flow chart. [file DataSheet_1.pdf]

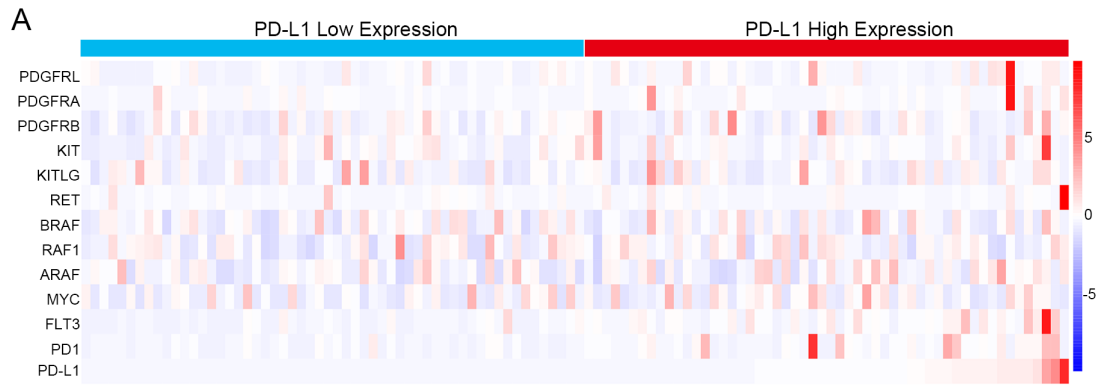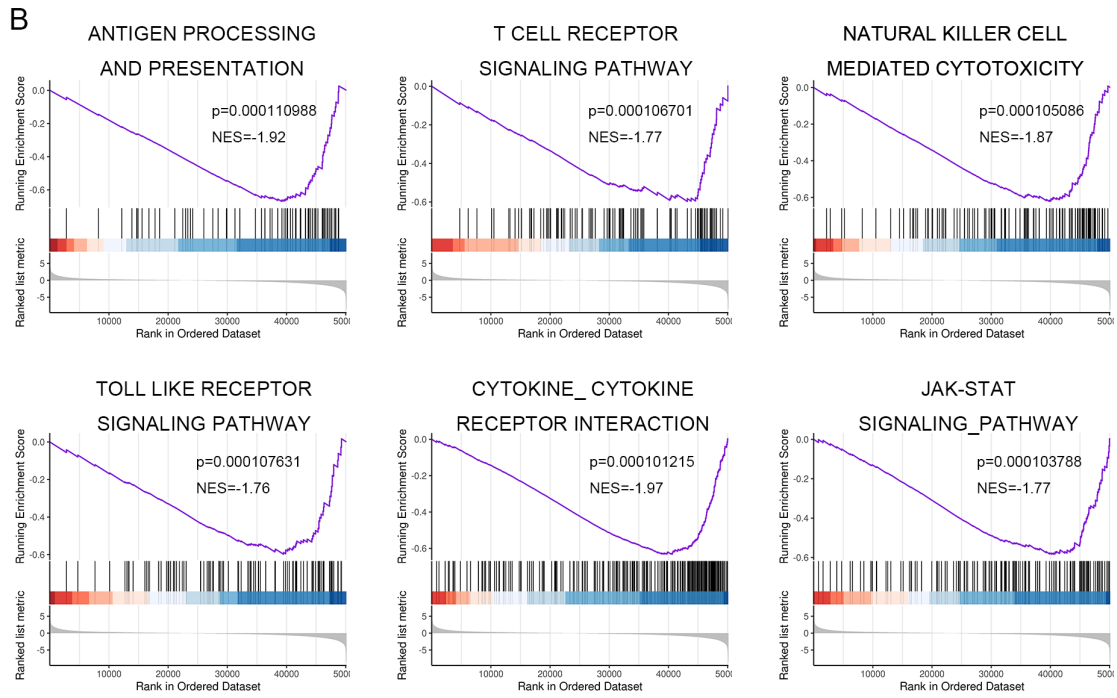

Supplement: Supplementary Figure 2 — Comparisons of RFS between patients with PD-L1 (≥3.0) and those with PD-L1 (<3.0) in 154 unselected HCC patients who received adjuvant sorafenib. [file DataSheet_2.pdf]

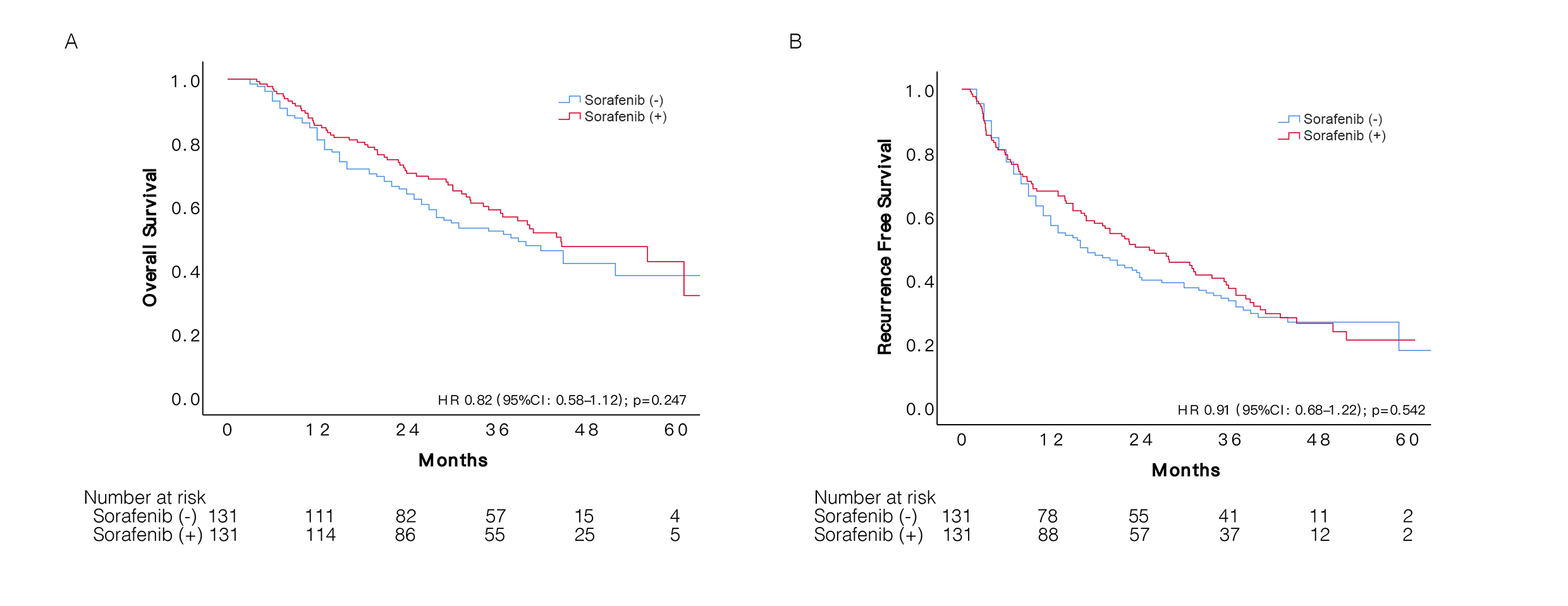

Supplement: Supplementary Figure 3 — Recurrence-free survival nomogram of 122 HCC patients who had adjuvant sorafenib. MAVI, macrovascular invasion; MIVI, microvascular invasion; PD-L1, programmed death-ligand 1; RFS, recurrence-free survival. [file Image_1.tif]

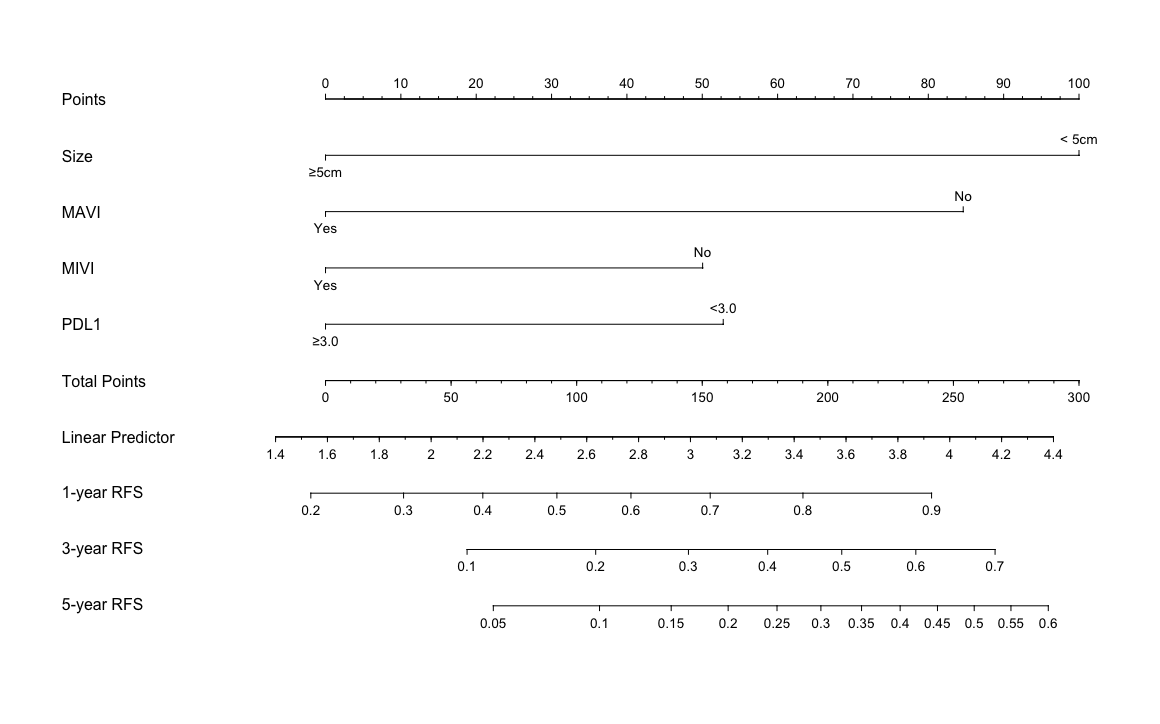

Supplement: Supplementary Figure 4 — Analysis of correlation between PD-L1 and sorafenib-related target molecules. (A) Heatmap showing relation between PD-L1 and other molecules; (B) GSEA analysis using KEGG gene set (C2). [file Image_2.tiff]
