# Supplementary material for: Overexpression of PD-L1 is an Independent Predictor for Recurrence in HCC Patients Who Receive Sorafenib Treatment After Surgical Resection
Source: Front Oncol. 2022 Jan 18;11:783335. doi: 10.3389/fonc.2021.783335 (PMC8804345; doi:10.3389/fonc.2021.783335)
Supplement: Supplementary file 5 [file Table_1.docx]

| Table S1. Tumor recurrence and salvage treatment in 122 patients with high risk of recurrence | | | |
| --- | --- | --- | --- |
|  | PD-L1(-) | PD-L1(+) | p |
|  | N=79 | N=43 |  |
| Recurrence, % | 52 (65.8) | 34 (79.1) |  |
| Recurrence within 2 years, % | 34 (65.4) | 26 (76.5) | 0.340 |
| Site of recurrence, % |  |  |  |
| Intrahepatic only, % | 39 (75) | 25 (73.5) | 0.823 |
| Extrahepatic only, % | 7 (13.5) | 6 (17.6) |  |
| Intra and extrahepatic, % | 6 (11.5) | 3 (8.8) |  |
| Number of recurrences, % |  |  |  |
| Solitary, % | 36 (69.2) | 23 (67.6) | 1.000 |
| Multiple, % | 16 (30.8) | 11 (32.4) |  |
| Salvage treatment, % |  |  |  |
| Liver transplantation, % | 2 (3.8) | 1 (2.9) | 0.972 |
| Liver resection, % | 14 (26.8) | 9 (26.5) |  |
| RFA, % | 8 (15.4) | 7 (20.6) |  |
| TACE, % | 22 (42.3) | 14 (41.2) |  |
| Others, % | 6 (11.5) | 3 (8.8) |  |

Abbreviation: RFA, Radiofrequency ablation; TACE, transcatheter arterial chemoembolization;
